# Single tablet regimens are associated with reduced Efavirenz withdrawal in antiretroviral therapy naïve or switching for simplification HIV-infected patients

**DOI:** 10.1186/1471-2334-14-26

**Published:** 2014-01-13

**Authors:** Massimiliano Fabbiani, Mauro Zaccarelli, Pierfrancesco Grima, Mattia Prosperi, Iuri Fanti, Manuela Colafigli, Alessandro D’Avino, Annalisa Mondi, Alberto Borghetti, Massimo Fantoni, Roberto Cauda, Simona Di Giambenedetto

**Affiliations:** 1 Institute of Clinical Infectious Diseases, Catholic University of Sacred Heart, Rome, Italy; 2 Viral Immunodeficiency Unit, National Institute for Infectious Diseases “Lazzaro Spallanzani”, Rome, Italy; 3 Infectious Diseases Unit, S. Caterina Novella Hospital, Galatina, Lecce, Italy; 4 Centre for Health Informatics, University of Manchester, Manchester, UK; 5 National Institute for Infectious Diseases “Lazzaro Spallanzani, Clinical Department, Via Portuense 292, 00149 Roma, Italy

**Keywords:** STR, Discontinuation, Combination antiretroviral therapy, Toxicity, Adherence

## Abstract

**Background:**

Efavirenz (EFV) administration is still controversial for its high rates of interruption mainly related to central nervous system side effects (CNS-SE). Aim of the study was to define if single tablet regimen (STR) as compared to bis-in-die (BID) or once-daily (OD) with ≥2 pills-a-day EFV formulations reduced the risk of interruption.

**Methods:**

Patients starting any cART regimen including EFV + 2NRTIs or switching to EFV + 2NRTIs for simplification after virological suppression were retrospectively selected. Incidence, probability and prognostic factors of interruption by different causes were assessed by survival analysis and Cox regression model.

**Results:**

Overall, 553 patients starting EFV-containing regimens were included: 38.2% started BID regimen, 44.5% OD regimens ≥2 pills and 17.4% STR. The overall proportion of EFV interruption was 37.4% at 4 years; at the same time point, interruptions for virological failure and toxicity were 8.8% and 16.5% (8% for CNS-SE), respectively. Starting EFV co-formulated in STR was associated with lower proportion of overall interruption at 4 years (17.1% vs. 40.6%, p < 0.01). Only one virological failure was observed with STR up to 4 years (1.1% vs. 10.3% in non-STR, p = 0.051). STR also accounted for lower proportion of interruption by patient decision (1.5% vs. 11.8%, p = 0.01). No differences of interruption by overall toxicity and CNS-SE were observed. In multivariable analysis, STR and male gender were associated with lower risk of EFV interruption, while higher CD4 nadir and IDU with higher risk.

**Conclusions:**

In our experience, starting EFV co-formulated in STR was associated with lower virological failure and higher adherence, despite a similar proportion of CNS toxicity, thus reducing the risk of treatment interruption.

## Background

Efavirenz (EFV) is a non-nucleoside reverse transcriptase inhibitor (NNRTI) which has shown good efficacy for the treatment of HIV infection [1-4]. In combination with a nucleoside reverse transcriptase (NRTI) backbone, it is currently recommended as a first line regimen in the treatment of therapy-naïve patients [5-8]. However, tolerability of this drug is still discussed due to potential toxicity/adverse effects, and in particular central nervous system side effects (CNS-SE).

It has been demonstrated that a substantial proportion of patients can experience CNS-SE during efavirenz treatment [9], and frequently CNS-SE can lead to regimen discontinuation [10]. Moreover, it has been suggested that long term treatment with EFV can contribute to the development of cognitive disorders in HIV-infected patients [11].

Nonetheless, several EFV characteristics can favor its large use: EFV has lower pill burden than protease inhibitors (PIs) and, in contrast to integrase inhibitor (InSTI) currently approved for first line therapy (Raltegravir), it can be administered once daily (OD). EFV formulation has been progressively improved during recent years from 200 mg capsules (which implied assumption of 3 capsules per day) to 600 mg tablets (one tablet OD) [12]. From 2008, EFV is also available in co-formulated shape with tenofovir and emtricitabine as a single tablet regimen (STR) [13]. STR combines a full antiretroviral regimen in one tablet taken once daily and this can be particularly convenient for HIV-infected patients. STR has demonstrated to improve patients’ satisfaction and adherence [14]. Finally, costs of EFV-based regimens are generally lower than those of PI- or InSTI-based first line regimens and are anticipated to be further reduced when generic formulations of EFV will be available.

As a consequence, more data on EFV use in the routine clinical setting are needed in order to better define the exact role of this drug in the treatment of HIV infection subject to its tolerability.

Therefore, aim of this study was to investigate rates and causes of discontinuation of EFV-based regimens in order to asses if better drug formulation up to STR reduced the risk of treatment interruption.

## Methods

### Patients included

This study included patients attending two clinical reference centers for HIV treatment in Italy (Catholic University of the Sacred Heart, Rome; Santa Caterina Novella Hospital, Galatina, Lecce). All patients signed a written, informed consent to be included in observational studies. This informed consent was approved by the local institutional Ethics Committees.

From the data-bases of the two centers, patients starting any combined antiretroviral regimen (cART) including EFV + 2 NRTIs were retrospectively selected from the date of EFV approval by the European Medicine Agency (1999) to March 2012. Patients were included in the analysis if they started an EFV-including cART for the first time (naïve patients) or switched to EFV + 2 NRTIs regimen for treatment simplification after virological suppression. Exclusion criteria were age less than 18 years, prior administration of mono/dual therapies, prior virological failure and prior NNRTIs use. Patients who previously failed treatment but without evidence of genotypic resistance mutations to any of the drugs in the prescribed regimen containing EFV - according to REGA rules [15] - were included.

Patients were followed from the time of EFV initiation (baseline) to discontinuation of the EFV-containing regimen or to the last available visit. Demographic, clinical and laboratory data, including virological and immunological assessment were collected both from recorded data and chart review.

Reasons for regimen switch or discontinuation, as reported by the caring physician and aggregated according to a pre-defined list [16] were collected and recorded in the database. In particular, our analysis was focused on the following causes of EFV discontinuation: virological failure, toxicity, CNS-SE, patient’s decision. Virological failure was considered a confirmed detectable HIV viral load (>50 copies/ml) at any point during follow-up.

### Statistical analysis

The probability of interruption by virological failure, toxicity, CNS-SE, patient’s decision and any cause was estimated by Kaplan Meier curves with log rank test for assessing differences in strata (e.g. STR compared with non-STR regimens). Follow-up time was carried out up to 48 months from EFV initiation, since STR containing EFV was available in Italy from 47 months. Observations were censored at the time of the last available visit or death, including patients lost in follow-up, who were classified as virological failure if the last HIV-RNA was detectable. Predictors of EFV discontinuation were explored by means of univariable/multivariable Cox proportional hazard regression. A statistical test was considered significant if the corresponding p-value was equal or less than 0.05. Analyses were performed using the IBM SPSS statistical package v. 20.0 (Armonk, NY; IBM Corp.).

## Results

A total of 553 patients starting EFV-containing regimens were included. Patient’s general characteristics are reported in Table 1. Of note, the proportion of therapy-naïve and therapy-switching patients was similar (51.2% vs. 48.8%). Most subjects switching to EFV + 2 NRTIs were from protease inhibitor-based regimen (527, 90.4%); 14 patients (5.2%) switched from 3 NRTI regimen, 9 (3.3%) from nevirapine-based regimen and 3 (1.1%) from raltegravir-based regimen: all subjects switched in order to reduce the number of pills or frequency of drug administrations.

**Table 1:** General characteristics of the population included in the study (n = 553)

	**N. patients (%)**	**Non-STR (n = 457)**	**STR (n = 96)**	**P value**
**Baseline calendar year**				
**1999**–**2003**	187 (33.8)	187 (40.9)	/	
**2004**–**2007**	181 (32.7)	181 (39.6)	/	
**2008-2012**	185 (33.5)	89 (48.1)	96 (100.0)	
**Age, years**^**a**^	39 (33–45)	38 (33–45)	42 (32–47)	0.06
**Male gender**	391 (70.7)	324 (70.9)	67 (69.8)	0.83
**Non-Italian nationality**	130 (23.0)	103 (23.4)	27 (28.1)	0.24
**Injecting drug use (as risk factor)**	93 (11.4)	57 (12.5)	6 (6.3)	0.08
**CDC class C**	130 (23.5)	109 (23.9)	21 (21.9)	0.68
**Years from HIV diagnosis**^**a**^	2.6 (0.7-7.4)	2.5 (0.5-7.4)	3.0 (1.4-7.6)	0.09
**Naïve**	283 (51.2)	248 (54.3)	35 (36.5)	**0.002**
**Number of treatment lines at EFV start**^**a,c**^	2 (2–3)	3 (2–3)	2 (2–3)	0.17
**Baseline Viral Load, log copies/mL**^**a,b**^	4.8 (4.6-5.2)	4.8 (4.6-5.3)	4.8 (4.1-5.3)	0.35
**Nadir CD4 cells count, cells/μL**^**a**^	196 (93–262)	180 (91–248)	246 (121–326)	0.1
**Baseline CD4 cells count, cells/μL**^**a**^	300 (207–437)	275 (185–433)	430 (322–573)	0.55
**Co-administered NRTIs:**				
**TDF + FTC/3TC**	315 (57.0)	219 (47.9)	96 (100.0)	
**ABC + 3TC**	21 (3.8)	21 (4.6)	/	
**AZT + 3TC**	130 (23.5)	130 (28.5)	/	
**Other**	87 (15.7)	87 (19.0)	/	

Notes: values are expressed as number (percentage), except for ^a^median (interquartile range); ^b^naïve patients; ^c^experienced patients. Bold values are statistically significant p values.

*Abbreviations:BID* twice daily, *OD* once daily, *STR* single tablet regimens, *EFV* efavirenz, *NRTIs* nucleoside reverse transcriptase inhibitors, *TDF* tenofovir, *FTC* emtricitabine, *3TC* lamivudine, *ABC* abacavir, *AZT* zidovudine: D4T, stavudine.

Overall, 38.2% started BID regimen, 44.5% OD regimens ≥2 pills and 17.4% STR. The most commonly prescribed NRTIs backbone was tenofovir + emtricitabine/lamivudine, accounting for more than half of the regimens included, followed by zidovudine + lamivudine; the combination abacavir + lamivudine had low frequency of prescription.

A total of 161 (29.1%) patients discontinued EFV during the 48 months follow up. The number of interruptions, the persons/years of follow-up and the incidence rates of interruption stratified by type of regimen are reported in Table 2. The rates of interruption were similar for bis-in-die (BID) and once-daily (OD) ≥2 pills/tablets regimens, while they were lower for STR.

**Table 2:** Probability of interruption by any cause by type of regimen started

	**N. (%)**	**Cumulative interruptions at 48 months (%)**	**Persons/years**	**Incidence rate (95%CI)**
**Overall**	553 (100.0)	161 (29.1)	12927	1.25 (1.07-1.45)
**Bis-in-die (BID)**	211 (38.2)	83 (39.3)	5939	1.40 (1.13-1.73)
**Once-daily (OD) >2 pills**	77 (13.9)	22 (28.6)	1640	1.34 (0.89-2.02)
**Once daily (OD) 2 pills**	169 (30.6)	42 (24.9)	3158	1.33 (0.99-1.79)
**Single tablet regimen (STR)**	96 (17.4)	14 (14.6)	2190	0.64 (0.38-1.07)

The main causes of EFV interruption were: CNS toxicity (n = 37, 6.7%); hypersensitivity (n = 15, 2.7%); metabolic toxicity (n = 11, 2.2%); other toxicity (n = 14, 2.5%); virological failure (n = 30, 5.4%); patient wish/non-compliance (n = 47, 7.9%). One patient died by non-HIV related causes during follow-up.

By Kaplan-Meier analysis, the estimated probabilities of EFV interruption were 19.1% at 1 year (95% confidence intervals, CI: 17.4-20.8) and 37.4% (95% CI: 34.9-39.9) at 4 years. Therapy-naïve patients had a higher probability of interruption as compared to patients switching therapy: 43.9% (95% CI: 40.3-47.5) vs. 30.0% (95% CI: 26.6-33.4) at 4 years (p = 0.013). Probabilities of interruptions for virological failure were 2.8% (95% CI: 2.0-3.6) and 8.8% (95% CI: 7.0-10.6), whilst those for toxicity were 10.2% (95% CI: 8.9-11.5) and 16.5% (95% CI: 14.5-18.5) at 1 and 4 years, respectively. CNS-SE accounted for about a half of interruptions for toxicity: 5.7% (95% CI: 4.6-6.8) and 8.0% (95% CI: 6.7-9.3), respectively. CNS-SE interruptions were generally observed in the first months of EFV use (only one interruption for CNS-SE was observed after the first 24 months). Probability of interruption for personal patient decision was 4.9% (95% CI 3.9-5.9) at 1 year and 10.3% (95% CI: 8.5%-12.1%) at 4 years.

**Figure 1:**
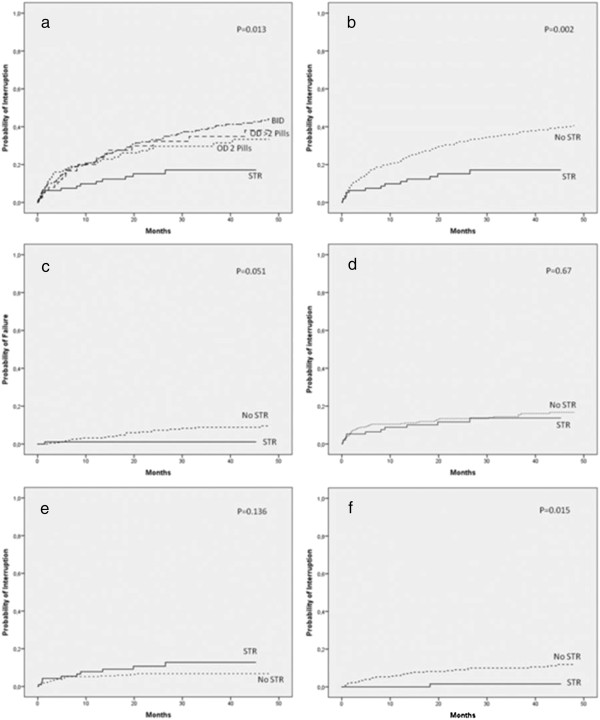
**a-f: Kaplan-Meier plots estimating probability of treatment interruption for specific reasons stratified by STR vs. no-STR.** Notes: **(1a)** overall probability of interruption by type of regimen; **(1b)** overall probability of interruption STR vs. no STR; **(1c)** probability of interruption by virological failure STR vs. no STR; **(1d)** probability of interruption by any side effect STR vs. no STR; **(1e)** probability of interruption by central nervous system side effects STR vs. no STR; **(1f)** probability of interruption by personal decision STR vs. no STR.

Figure 1a-f reports the survival analysis comparing STR vs. non-STR, stratified by reason of interruption. While no significant differences were observed comparing OD vs. BID regimens ≥2 pills/tablets (Figure 1a), STR were associated with a significant lower probability of overall interruption at 4 years: 14/96, 17.1% by Kaplan-Meier estimation (95% CI: 12.8-21.4) vs.147/457, 40.6% (95% CI: 37.9-43.3), p < 0.01 (Figure 1b). Only one virological failure (vs. 26) was observed with STR up to 4 years: 1.1% (95% CI: 0.0-2.2) vs. 10.3% (95% CI: 7.7-12.9), p = 0.051 (Figure 1c). No differences of interruption by overall toxicity and a higher, though non-significant, frequency of interruption by CNS-SE related to STR was observed (Figure 1d-e). In contrast, STR accounted for significant lower proportion of interruption by patient decision: 1 in STR patients (1.5%, 95% CI: 0.0-3.0) vs. 36 (11.8%, 95% CI: 9.8-13.8), (Figure 1f).

Table 3 reports the crude (univariable) and adjusted (multivariable) hazard ratios (HR) from fitting Cox regression for treatment interruption by any cause. At adjusted analysis STR and male gender were associated with lower risk of EFV interruption while intravenous drug use (IDU) as risk factor and higher CD4 nadir showed a higher risk. The association of interruption with higher CD4 nadir was detected, after adjustment in experienced patients (HR: 1.09, 95% CI: 1.02-1.18, p = 0.022) and not in naïve patients (HR: 1.03, 95% CI: 0.94-1.12, p = 0.54).

**Table 3:** Crude and adjusted hazard ratio (HR) of treatment interruption by Any Cause (Cox regression)

	**HR**	**95%CI**	**P**	**aHR**	**95%CI**	**p**
**Calendar year, per 1 year increase**	**0.95**	**0.91-0.99**	**0.021**	1.05	0.97-1.14	0.22
**Age, per 1 year increase**	0.99	0.97-1.00	0.11	1.00	0.98-1-01	0.61
**Male gender**	**0.67**	**0.49-0.93**	**0.015**	**0.68**	**0.50-0.95**	**0.022**
**Non-Italian nationality**	1.30	0.92-1.85	0.13	1.44	0.98-2.11	0.06
**IDU (as risk factor)**	**1.78**	**1.19-2.66**	**0.005**	**1.98**	**1.27-3.09**	**0.003**
**CDC class C**	0.90	0.62-1.32	0.60	1.02	0.67-1.55	0.92
**Years from HIV diagnosis (per 1 year increase)**	1.00	0.97-1.03	0.94	1.00	0.97-1.03	0.98
**Naive (vs. switched)**	**1.49**	**1.09-2.04**	**0.013**	1.31	0.74-2.32	0.36
**Number of treatment lines at EFV start, per 1 year increase**	0.92	0.78-1.07	0.26	1.19	0.95-1.47	0.13
**Baseline Viral Load, per 1 log increase**	1.11	0.95-1.29	0.31	1.06	0.92-1.23	0.43
**Nadir CD4, per 50 cells/μL increase**	1.04	0.99-1.10	0.06	**1.13**	**1.04-1.24**	**0.005**
**Baseline CD4, per 50 cells/μL increase**	0.97	0.93-1.01	0.09	0.95	0.89-1.01	0.12
**Backbone:**						
**TDF + FTC/3TC**	Ref			Ref		
**ABC + 3TC**	0.76	0.28-2.08	0.59	0.61	0.22-1.70	0.34
**AZT + 3TC**	**1.53**	**1.07-2.20**	**0.021**	1.50	0.91-2.47	0.11
**Other**	**1.65**	**1.10-2.47**	**0.015**	1.69	0.95-3.02	0.08
**STR**	**0.43**	**0.25-0.75**	**0.003**	**0.43**	**0.23-0.83**	**0.012**

*Abbreviations:HR* hazard ratio, *CI* confidence intervals, *IDU* injecting drug users, *TDF* tenofovir, *FTC* emtricitabine, *3TC* lamivudine, *ABC* abacavir, *AZT* zidovudine, *STR* single tablet regimen.

Age was not associated with treatment interruption, even taking into account subgroups of patients: i.e. older age (>50 years) was not associated with EFV interruption.

We also performed different sets of sensitivity analyses in order to better account for the imbalance in some characteristics of the two groups at baseline. Excluding patients not treated with a tenofovir-containing backbone and then comparing STR vs. OD-2 pills regimens (tenofovir/emtricitabine as backbone) (249 patients), a lower proportion of treatment interruption in STR group was detected (17.1% vs 34.7%, p = 0.022) at survival analysis. Moreover, excluding patients with IDU as risk factor (490 patients), a lower proportion of interruption was also detected (15.3% vs. 37.9%, p = 0.005) and finally, taking into account patients starting EFV-containing regimen from 2007 (since STR was available in Italy, 213 patients), still a significant lower proportion of interruption was observed (17.1% vs 34.1%, p = 0.03).

## Discussion

EFV has been available for clinical use for many years and there is a large experience with the drug administration in clinical practice. However, studying EFV and its formulation history is still of clinical relevance, because of complex mechanics of tolerability and side effects, and also because its patent is next to expire. A recent study focused on the high risk of EFV interruption, mainly related to CNS-SE [17], but there is a lack of comprehensive works.

Our data showed that the probability of EFV interruption (nearly 30% after 48 months) was similar to that observed in Italy at the beginning of the EFV availability [18]. However, we also observed that the improved EFV formulation had a positive effect on the risk of interruption. In particular, the effect was minimal and not statistically significant when moving from BID to OD regimens or reducing the number of pills/tablets up to 2 within OD regimens. Conversely, the real significant improvement was observed when taking into account STR, which demonstrated a clear advantage also compared to 2 pills regimens.

The greatest improvement in the risk of interruption related to STR was evident when considering virological failure and personal patient decision as reasons of interruption, suggesting in both cases an improvement in patients’ adherence. The adherence improvement related to the switch from various types of regimens to STR had already been described [19], leading to a lower probability of virological failure [20]. A clear improvement in adherence and quality of life related to the switch to STR in patients previously treated with OD regimens containing EFV has been suggested [21].

Our data also demonstrated that the use of STR was not associated with lower probability of interruption for CNS-SE and that patients preferred the simplest regimen in spite of non-apparent differences in toxicity.

These results are interesting in light of the expiration of EFV patent in 2013, as well as that of other antiretroviral drugs (e.g. lamivudine, abacavir), considered a favorable circumstance to exploit the development of generic antiretroviral drugs to reduce costs [22].

The use of generic drugs may have as consequence relevant changes in formulations, such as the reversion from STR to ≥2 pills/tablets combination. Possible consequences of losing STR in terms of adherence and treatment success have been already focused [23]. It is therefore important to emphasize that this choice must be evaluated in light of its real cost-effectiveness, taking into account the increased risk of interruption and virological failure. Our findings demonstrate that starting regimens with a higher pill burden can be associated with a higher risk of treatment interruption, mainly related to adherence, and thus suggesting the benefit of maintaining STR. However, it should also be emphasized that not all patients tolerating well a STR will be certainly less adherent or will interrupt treatment after switching to a regimen with the same drugs, not co-formulated. The choice of the regimen should be tailored on individual patients, discussing benefit and potential risk of each decision.

In our analysis higher CD4 nadir was associated with higher risk of interruption. This association was detected only in the multivariable analysis, and can be interpreted with higher adherence in patients who achieved lower CD4 level, in particular experienced patients, in whom a low CD4 nadir at baseline was observed.

We acknowledge that our study can have some limitations because uncontrolled biases can occur in retrospective studies; however, reasons for regimen switch or discontinuation were reported by the caring physician and aggregated according to a pre-defined list at the time of occurrence, similarly to other large observational multicenter databases. The two groups of patients (STR and non-STR) were not completely matched for all characteristics at baseline; in particular, a higher proportion of naïve patients and a higher, despite not significant, percentage of IDU was observed in the non STR group. This could have partly influenced the results; however, all these variables were adjustment factors in the multivariable model and several subgroup sensitivity analysis were performed to account for this potential bias. Another potential limitation of the present analysis may be related to differences in regimens prescribed by calendar year. Indeed, STR was approved in Italy from 2008, but non-STR regimens are currently prescribed when starting an EFV-containing regimen. However, in our analysis, calendar year, IDU and CD4 count/CD4 nadir were used as adjustment factors to correct this possible bias.

## Conclusions

In conclusion, our results demonstrated that starting EFV co-formulated in STR was associated with lower hazard of virological failure and with higher adherence as compared to other formulations (i.e. BID or ≥2 pills OD), despite keeping similar toxicity profiles and in particular CNS toxicity, with subsequent lower risk of overall treatment interruption.

## Abbreviations

STR: Single tablet regimen.

## Competing interests

MF received speakers’ honoraria from Abbott Virology, Merck Sharp & Dohme and Janssen-Cilag. MZ received speakers’ honoraria from Abbott Virology, Gilead Science, Merck Sharp & Dohme and Janssen-Cilag. MP is supported by University of Manchester’s Health Research Center (HeRC) under the Medical Research Council grant MR/K006665/1. MC has been a paid consultant for Merck Sharp & Dohme, Italy and has been employed by Bristol-Myers-Squibb, Italy since May 10th, 2010 to Feb 28th 2011. RC has been advisor for Gilead and Janssen-Cilag, received speakers’ honoraria from ViiV, Bristol-Myers Squibb, Merck Sharp and Dohme and Janssen-Cilag, and research support from “Fondazione Roma”. SDG received speakers’ honoraria and support for travel meetings from Gilead, Bristol-Myers Squibb, Abbott, Boehringer Ingelheim, Janssen-Cilag, and GlaxoSmithKline. All the other authors have nothing to declare.

## Authors’ contributions

MF contributed to study design, data interpretation and article writing; MZ contributed to study design, data interpretation/analysis and article writing; PG, IF, MC, AD, AM, AB contributed to data collection; MP contributed to data interpretation and analysis; MF, RC and SDG coordinated the project and contributed to the interpretation of data. All authors reviewed the manuscript during preparation, provided critical feedback and approved the final manuscript.

## Pre-publication history

The pre-publication history for this paper can be accessed here:

http://www.biomedcentral.com/1471-2334/14/26/prepub
